# Case report: Helical tomotherapy-based whole lung irradiation with simultaneous integrated boost for pulmonary metastatic Ewing’s sarcoma: a case series report and feasibility analysis

**DOI:** 10.3389/fonc.2026.1823784

**Published:** 2026-05-20

**Authors:** YuanYou Yang, Lu Xie, Jie Xu, Xin Sun, Gang Ren

**Affiliations:** 1 Department of Radiotherapy, Peking University Shougang Hospital, Beijing, China; 2 Musculoskeletal Tumor Center, Peking University People’s Hospital, Beijing, China

**Keywords:** case series, Ewing’s sarcoma, helical tomotherapy, pulmonary metastases, radiation pneumonitis, whole-lung irradiation

## Abstract

**Background:**

Ewing’s sarcoma (EwS) frequently metastasizes to the lungs. While whole-lung irradiation (WLI) improves survival, conventional techniques carry a risk of cardiac toxicity. Helical Tomotherapy (HT) offers superior target conformity and the ability to deliver a simultaneous integrated boost (SIB) to metastases. This case series evaluates the feasibility and short-term outcomes of HT-based WLI with SIB in patients with pulmonary metastatic EwS, with a focus on the challenges encountered across a variety of complex clinical scenarios.

**Case description:**

We report on five patients (3 male, 2 female; age range 15–36 years, mean age 24.2 years) with pulmonary/pleural metastases who received HT-based WLI (12–15 Gy/10 fractions) with SIB to thoracic lesions (27–45 Gy). The cases are unique in their diversity: one patient with 36 bilateral lung metastases achieving complete response (Case 2), one with prior sternal SBRT developing grade 3 radiation pneumonitis (RP) on concurrent immunotherapy (Case 5; thoracic radiotherapy delivered to spatially distinct, non-overlapping target volumes), and one with pleural progression after multiple chemotherapy lines (Case 3). Concurrent therapies included anlotinib (n=4), sintilimab (n=1), or chemotherapy (n=1). With a median follow-up of 5.5 months (range 2-12.5), the overall response rate was 86.7% (59/68 lesions: 53 complete responses, 1 near-CR, 5 partial responses). Median lung/pleural progression-free survival was 5.5 months, with four patients surviving and one dying from extrapulmonary progression. Acute toxicity was manageable, including one case of grade 3 RP (resolved with steroids). Mean heart dose was 7.39 ± 0.81 Gy and lung V20 was 19.15 ± 5.03%. All plans achieved satisfactory target coverage with D95% ≥ prescription dose for both PTVall and PTVp.

**Conclusions:**

HT−based WLI with SIB provides excellent target coverage and satisfactory OAR sparing, is feasible and adaptable in complex settings, and achieves encouraging short−term local control in pulmonary metastatic Ewing’s sarcoma; caution is warranted regarding radiation pneumonitis risk with concurrent immunotherapy.

## 1 Introduction

Ewing’s sarcoma (EwS), the second most common malignant bone tumor in children and adolescents, is highly sensitive to chemotherapy and radiotherapy (1, 2). The lung is the predominant site of metastasis, occurring in approximately 50-60% of patients with disseminated disease, which portends a poor prognosis (3, 4). Whole-lung irradiation (WLI) has been established as a crucial component of multimodality treatment, significantly reducing pulmonary recurrence and improving event-free and overall survival (5–13), and is recommended by current guidelines (14).

Conventional anteroposterior (AP/PA) WLI techniques deliver significant cardiac dose, associated with increased risk of congestive heart failure and other late effects, particularly in young patients (15, 16). As an advanced photon radiotherapy approach, helical tomotherapy (HT) provides superior dose conformity and more effective cardiac sparing compared with conventional 3D−CRT, static IMRT, and VMAT techniques in whole−lung or thoracic irradiation, as validated in multiple dosimetric comparison studies (17–19). Proton therapy can achieve further cardiac sparing (17), however, its availability remains limited and less widespread compared with photon-based radiotherapy techniques. Helical tomotherapy (HT) offers superior dose conformality for complex targets like the lungs and enables simultaneous integrated boost (SIB) to multiple metastatic lesions (17). What makes this case series unique is the application of HT-WLI with SIB across diverse clinically challenging scenarios, including extensive miliary pulmonary metastases, prior thoracic radiotherapy, pleural-predominant chemorefractory disease, isolated lung recurrence, and combination treatment with targeted therapy or immunotherapy. This series demonstrates the versatility of HT-WLI-SIB and provides preliminary safety and efficacy data across these complex presentations.

## 2 Case description

### 2.1 Study design and patient selection

This retrospective, single-center case series included eligible patients treated between October 2024 and June 2025 at a tertiary care medical center. Inclusion criteria were: (1) histologically confirmed EwS; (2) radiologically diagnosed lung and/or pleural metastases or recurrences; (3) Eastern Cooperative Oncology Group performance status (ECOG PS) 0-2; and (4) unresectable thoracic lesions or patient refusal of surgery. Exclusion criteria were: (1) ≥2 prior courses of thoracic radiotherapy; (2) moderate-to-severe pulmonary dysfunction or active pulmonary infection; (3) severe hepatic or renal impairment; or (4) pregnancy. The primary aim of this study was to evaluate the feasibility of HT−based WLI with SIB for pulmonary metastatic Ewing’s sarcoma. Feasibility was defined by four prespecified criteria: technical deliverability of treatment plans, adequate target volume coverage, effective organ−at−risk sparing, and acceptable acute toxicity.

### 2.2 Case summaries and timeline

**Figure 1:**
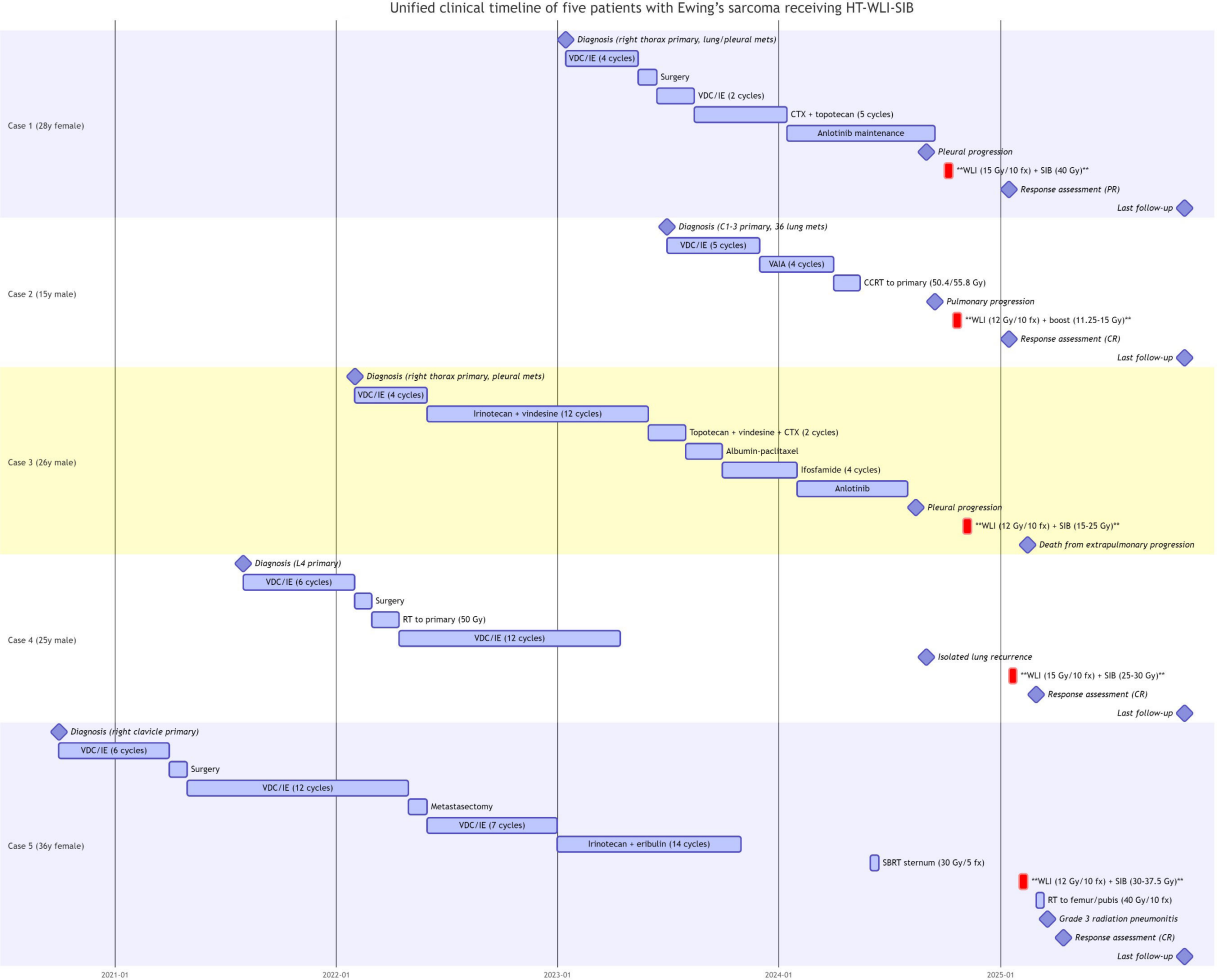
Unified clinical timeline for five patients with Ewing’s sarcoma receiving HT-WLI-SIB. Each row represents an individual patient’s journey from initial diagnosis to last follow-up. Key events include: lines of systemic therapy (colored bars), radiotherapy courses (blue rectangles with dose/fractionation), metastatic events (red diamonds), and responses (green, complete response; yellow, partial response/stable disease). The vertical dashed line marks the start of HT-WLI. CR, complete response; PR, partial response; SD, stable disease; PD, progressive disease; SBRT, stereotactic body radiation therapy; WLI, whole lung irradiation; mo, months.

**Figure 1** presents a unified timeline of the clinical course for all five patients from initial diagnosis through last follow-up.

#### 2.2.1 Case 1

A 28-year-old female, ECOG PS 0 with no respiratory distress or abnormal chest findings, presented with T2N0M1 EwS (right thorax primary) with lung/pleural metastases. Prior therapies included 4 cycles VDC/IE (vincristine, doxorubicin, cyclophosphamide alternating with ifosfamide, etoposide) → surgery → 2 cycles VDC/IE → 5 cycles cyclophosphamide + topotecan → anlotinib maintenance. Pre-WLI status showed progressive pleural metastases. She received WLI (15 Gy/10 fractions) + sequential SIB to 8 pleural lesions (total 40 Gy) with concurrent anlotinib. Unique aspect: Isolated pleural metastases responding to combined anlotinib and radiotherapy without pulmonary parenchymal involvement.

#### 2.2.2 Case 2

A 15-year-old male, ECOG PS 1 with mild exertional dyspnea, presented with T4aN1M1 EwS (cervical spine C1–3 primary) with 36 bilateral lung metastases. Prior therapies: 5 cycles VDC/IE + 4 cycles VAIA (vincristine, doxorubicin, ifosfamide, dactinomycin) → concurrent chemoradiotherapy to primary (PTV 50.4 Gy, GTV 55.8 Gy). Pre-WLI showed progressive pulmonary metastases. He received WLI (12 Gy/10 fractions) with sequential local boost (11.25 Gy/5 fractions to PTVp, 15 Gy/5 fractions to GTVp) to 24 residual lesions. Unique aspect: Extremely high burden of miliary disease (36 lesions) with complete response to relatively low-dose radiation, demonstrating high radiosensitivity of EwS, consistent with previous reports in the literature (20).

#### 2.2.3 Case 3

A 26-year-old male, ECOG PS 1 with clear breath sounds and no respiratory distress, presented with T3N1M1 EwS (right thorax primary). Prior therapies: 4 cycles VDC/IE → 12 cycles irinotecan + vindesine → 2 cycles topotecan + vindesine + cyclophosphamide → albumin-paclitaxel → 4 cycles ifosfamide → anlotinib. Pre-WLI showed progressive pleural metastases. He received WLI (12 Gy/10 fractions) with sequential SIB to pleural lesions (15–25 Gy boost). Unique aspect: Chemorefractory pleural-only progression with short interval to death from extrapulmonary disease, highlighting the highly aggressive clinical behavior of chemorefractory EwS.

#### 2.2.4 Case 4

A 25-year-old male, ECOG PS 0 with no abnormal pulmonary or neurological findings, presented with initial T4aN0M0 EwS (L4 primary) who developed isolated lung recurrence 30 months post-diagnosis. Prior therapies: 6 cycles VDC/IE → surgery → radiotherapy to primary (50 Gy) → 12 cycles VDC/IE. He received WLI (15 Gy/10 fractions) with sequential SIB (PTVp 25 Gy/5 fractions, GTVp 30 Gy/5 fractions) and concurrent VDC/IE chemotherapy. Unique aspect: It represented a rare case of isolated lung recurrence of Ewing’s sarcoma, which obtained satisfactory local control following combined WLI and concurrent chemotherapy.

#### 2.2.5 Case 5

A 36-year-old female, ECOG PS 1 with mild exertional dyspnea but no resting hypoxemia, presented with initial T2N0M0 EwS (right clavicle primary) who developed widespread metastases (nodes, lung, pleura, bone). Prior therapies: 6 cycles VDC/IE → surgery → 12 cycles VDC/IE → metastasectomy → 7 cycles VDC/IE → 14 cycles irinotecan + eribulin → SBRT to sternum (30 Gy/5 fractions). Critically, she had received prior sternal SBRT 8 months before WLI. She received WLI (12 Gy/10 fractions) + SIB to left pleura/sternum/humerus (30-37.5 Gy/10 fractions), followed by 40 Gy/10 fractions to femoral/pubis lesions, with concurrent anlotinib and sintilimab. Unique aspect: thoracic radiotherapy delivered to spatially distinct, non-overlapping target volumes, combined with dual targeted/immunotherapy, resulting in grade 3 radiation pneumonitis.

## 3 Diagnostic assessment and therapeutic intervention

### 3.1 Radiotherapy planning and delivery

All patients were immobilized using vacuum cushions and thermoplastic masks. Contrast-enhanced computed tomography (CT) simulation was performed with 3-mm slice thickness. 4DCT simulation was not performed; respiratory motion was mitigated using abdominal compression, and a standardized 5−mm PTV margin was applied to account for residual target displacement, which is sufficient for young patients with limited respiratory motion. For Cases 1, 3, and 5, magnetic resonance imaging was co-registered with planning CT for target delineation.

Target volumes were defined as:

- Gross Tumor Volume (GTV): All radiologically visible thoracic disease.
- Clinical Target Volume for metastases (CTVp): GTV + 5–10 mm margin, edited to respect anatomical boundaries.
- Planning Target Volume for metastases (PTVp): CTVp + 5 mm isotropic expansion.
- CTVall: Whole lung plus all metastatic lesions (CTVp).
- PTVall: CTVall + 5 mm expansion.

**Table 1** details the individualized target volume definitions and prescription doses for each patient. Treatment was delivered in two sequential phases using HT. Course 1 comprised WLI to PTVall, with SIB to thoracic GTVs delivered only in Case 5; all other patients received sequential focal boost to residual or high-risk lesions in Course 2. Organ-at-risk constraints were derived from QUANTEC protocols (18, 19, 21, 22). Daily megavoltage CT guidance was used for setup verification.

**Table 1:** Individualized target volume definitions and prescription doses.

NO.	Definition of target volume (unique aspects only)	First course dose	Second course dose	Concurrent treatrment
1	GTVp: pleural metastases; CTVall: whole lung + CTVp	PTVall:15Gy/10Fx	PTVp: 11.25 Gy/5 Fx; GTVp: 15 Gy/5 Fx (sequential boost)	Anlotinib
2	GTVp: lung metastases (36 lesions); CTVall: whole lung only	PTVall: 12Gy/10Fx	PTVp: 11.25 Gy/5 Fx; GTVp: 15 Gy/5 Fx (sequential boost)	Anlotinib
3	GTVp: pleural metastases + primary; CTVall: whole lung + CTVp; boost1/boost2 (5 mm retraction)	PTVall: 12Gy/10Fx	GTVp: 15 Gy/5 Fx; GTVp boost1: 20 Gy/5 Fx; GTVp boost2: 25 Gy/5 Fx (sequential boost)	Anlotinib
4	GTVp: lung metastases (2 lesions); CTVall: whole lung only	PTVall:15Gy/10Fx	PTVp: 25 Gy/5 Fx; GTVp: 30 Gy/5 Fx (sequential boost)	VDC/IE
5	GTVp: sternum, humerus, nodes, lung, pleura; GTVbone: femurs + pubis; CTVall: whole lung + CTVp	PTVall: 12 Gy/10 Fx; GTVp boost1: 30 Gy/10 Fx; GTVp boost2: 37.5 Gy/10 Fx (simultaneous integrated boost, SIB)	PTVbone: 40 Gy/10 Fx	Anlotinib and Sintilimab

Margins: CTVp, GTVp +5−10 mm (5 mm near OARs); PTV, CTV +5 mm. Boost: Sequential (Cases 1−4) or simultaneous integrated boost – SIB (Case 5). For Case 3, boost1/boost2 indicate stepwise escalation. Abbreviations: GTV, gross tumor volume; CTV, clinical target volume; PTV, planning target volume; CTVall, whole lung (± CTVp); SIB, simultaneous integrated boost; VDC/IE, chemotherapy regimen.

### 3.2 Dosimetric assessment

**Table 2** summarizes dosimetric parameters for target coverage and organs at risk (OARs), presented separately for WLI alone and the combined WLI + boost plan. For OARs, all dosimetric parameters were within the safe constraints recommended by QUANTEC guidelines, confirming effective cardiac and normal tissue sparing.

**Table 2:** Dosimetric parameters for target coverage and organs at risk in five patients receiving HT-WLI-SIB.

Parameter	Whole lung irradiation alone		Whole lung irradiation + Boost	
	Mean ± SE	Median (range)	Mean ± SE	Median (range)
PTVall D95%(Gy)	13.26 ± 0.51	13.42 (12.14–14.68)	13.52 ± 1.46	13.87 (12.05–15.92)
PTVall V100% (%)	98.2 ± 1.1	98.7 (96.2–99.5)	98.6 ± 1.2	99.0 (96.8–99.8)
PTVp D95% (Gy)	–	–	36.84 ± 5.22	37.12 (27.44–44.89)
PTVp V100% (%)	–	–	97.9 ± 1.5	98.3 (95.1–99.4)
Heart Dmax (Gy)	15.58±1.62	14.97 (12.51-21.45)	24.71 ± 0.96	25.49 (21.45-27.04)
Heart Dmean (Gy)	7.39 ± 0.81	7.25 (5.81-10.43)	11.11 ± 1.01	11.66 (7.75-13.95)
Lung all Dmax (Gy)	19.68 ± 4.86	16.71 (12.79-38.79)	36.21 ± 3.92	32.45 (28.54-50.38)
Lung all Dmean (Gy)	14.19 ± 0.80	14.27 (12.28-16.15)	18.10 ± 1.38	17.35 (14.27-22.44)
Lung all V20 (%)	0	0	19.15 ± 5.03	21.47 (3.90-30.39)
Spinal cord Dmax (Gy)	10.67 ± 1.25	9.39 (8.65-15.51)	16.76 ± 2.64	18.25 (9.39-23.85)
Esophagus Dmax (Gy)	14.06 ± 1.06	12.69 (12.02-17.43)	20.65 ± 1.55	21.83 (16.50-24.05)
Esophagus Dmean (Gy)	9.51 ± 0.38	9.81 (8.36-10.49)	12.51 ± 1.25	14.04 (8.97-14.89)
Thyroid Dmax (Gy)	3.27 ± 0.38	3.36 (2.29-4.49)	4.55 ± 1.01	3.41 (2.29-7.64)
Thyroid Dmean (Gy)	1.34 ± 0.16	1.47 (0.7-1.59)	4.56 ± 2.78	2.17 (0.70-15.66)

**Figure 2:**
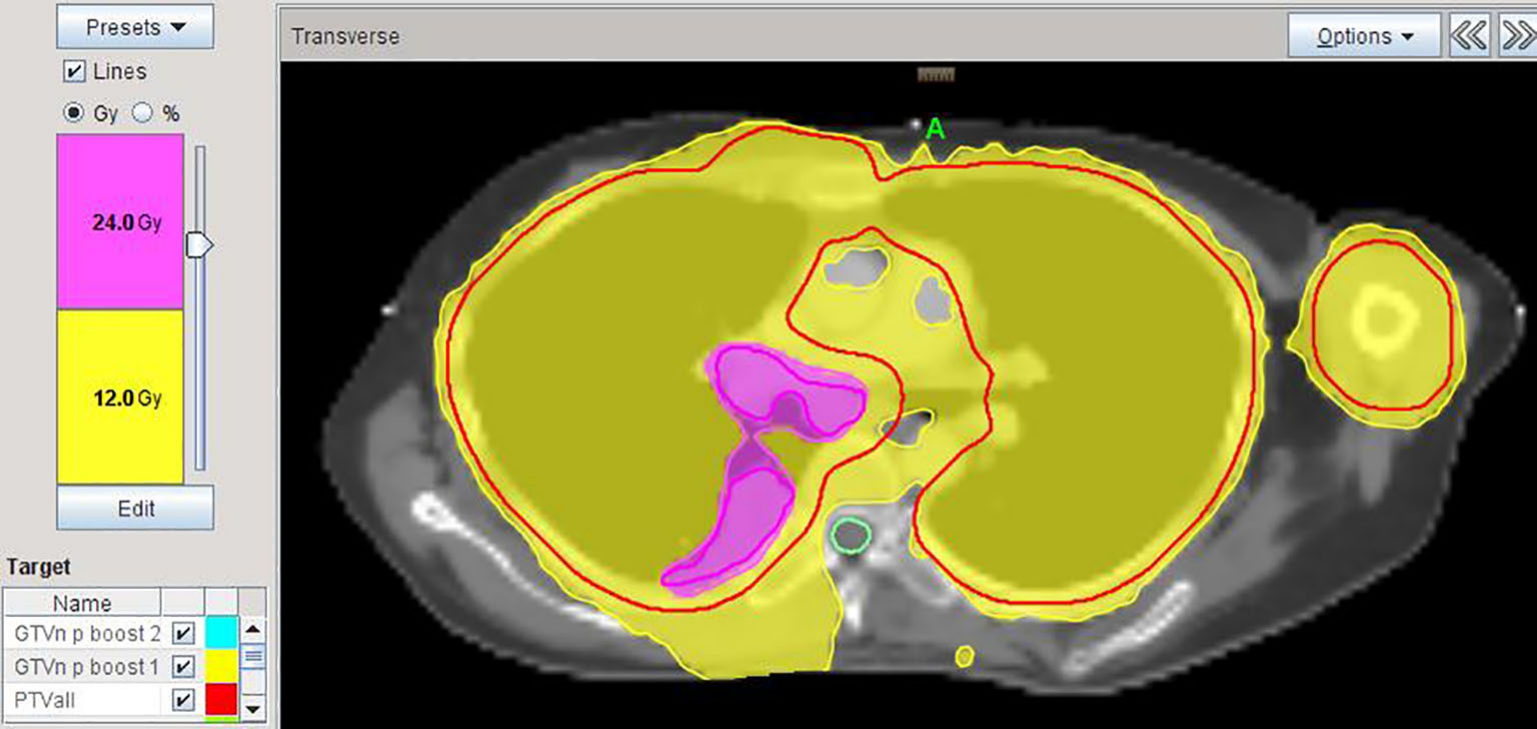
Dose distribution of whole-lung irradiation (12 Gy/10 Fx) with simultaneous integrated boost to the complex-shaped right hilar lesion, mediastinal lymph nodes, and right pleural lesion up to 24 Gy/10 Fx (double dose) in Case 5.

All patients successfully completed treatment with satisfactory target coverage, favorable OAR dosimetry (**Table 2**), and manageable acute toxicities (**Table 3**), thus meeting all predefined feasibility endpoints. **Figure 2** shows the dose distribution of whole-lung irradiation at 12 Gy/10 Fx with simultaneous integrated boost to the complex-shaped right hilar lesion, mediastinal lymph nodes, and right pleural lesion up to 24 Gy/10 Fx (double dose) in Case 5.

**Table 3:** Individual patient tumor response, toxicity, and survival outcomes.

NO.	Local tumor efficacy	Acute RP	Systemic tumor status at the last follow-up	Status of survival
1	Right pleural lesions (8): CR 6,PR 2	NO	New mediastinal LN metastasis at 7 months post-WLI	Alive
2	Intrapulmonary lesions (36): CR 36	NO	No new metastatic tumor occurred at 10.5 months post-WLI	Alive
3	Right pleural lesions (5): PR 1, SD 4 Primary tumor lesion (1):SD 1	NO	New retroperitoneal lymph node metastases and diffuse bone marrow metastases at 1.5 months post-WLI	Death
4	Intrapulmonary lesions (2): CR 2	NO	No progression at 5.5 months post-WLI	Alive
5	intrapulmonary lesions (6): CR 6 Hilar lymph node lesions (1): CR 1 Mediastinal lymph nodes lesions (1): CR 1 Pleural lesions (3): CR 1, near CR 1, PR 1 Sternum lesions (1): PR 1 left humerus lesions (1): SD 1 Left femur lesions (1): SD 1 Right femur lesions (1): SD 1 Left pubic lesions (1): SD 1	Grade 3	No progression at 5 months post-WLI	Alive

CR, complete response; near CR, near complete response ; PR, partial response; SD, stable disease; RP, radiation pneumonitis; LN, lymph node.

## 4 Follow-up and outcomes

### 4.1 Tumor response

With median follow-up of 5.5 months (range 2-12.5), 68 thoracic lesions were evaluable. Overall response rate was 86.7% (59/68), comprising 53 complete responses (CR), 1 near-CR, and 5 partial responses (PR). Disease control rate was 100%.

**Figure 3:**
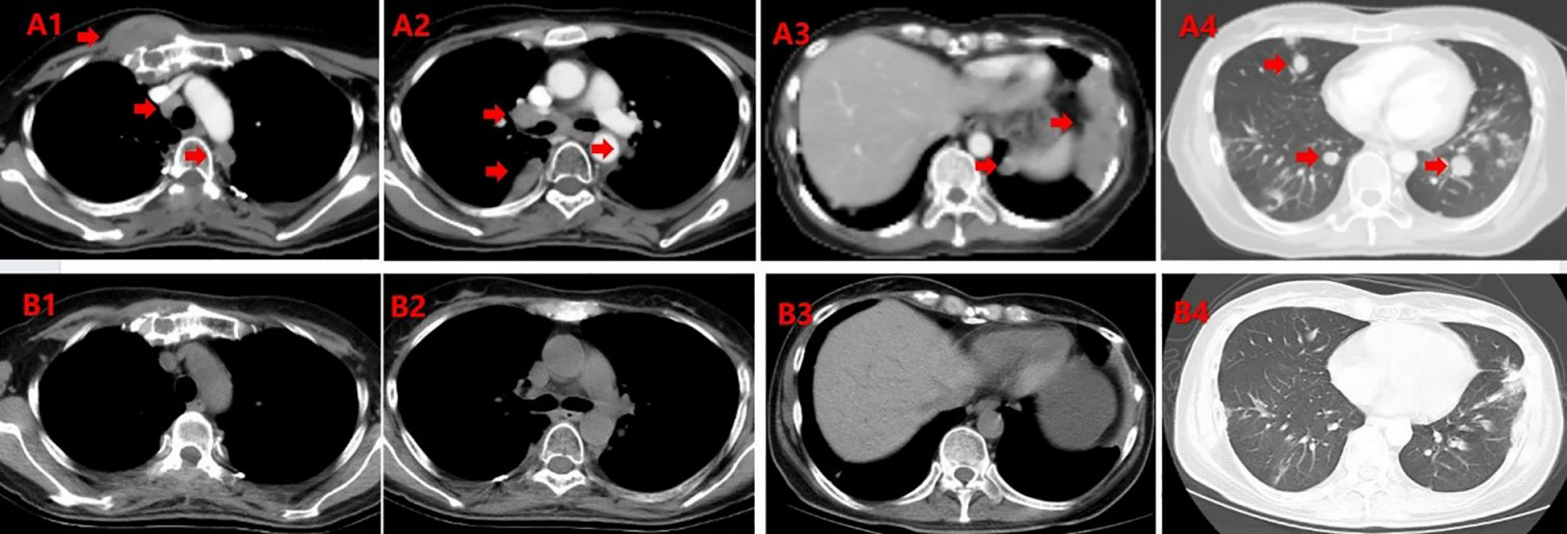
Comparative imaging of Patient 5 before and 1.5 months after HT-WLI-SIB. Pre-RT CT images **(A1–A4)** and post-RT CT images **(B1–B4)** show: **(B1)** Complete response of mediastinal lymph nodes and left pleural lesions; partial response of sternal metastasis. **(B2)** Complete response of hilar lymph nodes; near-complete response of right pleural lesion. **(B3)** Partial response of left pleural lesion; complete response of left pulmonary metastasis. **(B4)** Complete response of pulmonary metastases.

**Table 3** details individual patient responses and outcomes. **Figure 3** shows representative imaging from Patient 5 demonstrating CR of mediastinal nodes and pulmonary metastases at 1.5 months post-WLI.

### 4.2 Toxicity

Acute toxicity was generally mild. One patient (Case 4) developed grade 3 thrombocytopenia (platelets 27×10^9^/L) during concurrent VDC/IE, resolving after 2-week treatment break and thrombopoietin.

The most clinically significant toxicity was grade 3 radiation pneumonitis in Patient 5, occurring 1 month post-WLI. She developed dyspnea, dry cough, and radiation-field ground-glass opacities on chest CT. Notably, her lung mean dose and V20 remained well within QUANTEC dose constraints, suggesting that concurrent administration of anlotinib and sintilimab may have contributed to the occurrence of pulmonary toxicity. Symptoms resolved within 3 days of initiating corticosteroids (methylprednisolone 1 mg/kg/day with rapid taper). No grade ≥2 RP occurred in other patients.

No significant differences were observed in pre- and post-WLI complete blood counts (WBC: p=0.88; platelets: p=0.16).

## 5 Discussion

### 5.1 Strengths and limitations of the approach

This case series suggests the potential feasibility of HT-based WLI with SIB across five clinically challenging scenarios in metastatic EwS. The primary strength is the demonstration of technique adaptability: (1) in miliary disease (Case 2, 36 lesions) achieving CR with relatively low doses, consistent with the high radiosensitivity of EwS as documented in the literature (20); (2) in thoracic radiotherapy delivered to spatially distinct, non-overlapping target volumes (Case 5), where HT enabled conformal avoidance of high-dose overlap regions; (3) in pleural-only disease (Cases 1, 3), where HT’s ability to sculpt dose to the pleural surface was advantageous; (4) in combination with modern systemic therapies (anlotinib, sintilimab), providing preliminary safety data; and (5) in patients with multiple metastatic lesions and complex target configurations (Case 5). The satisfactory target coverage further validates that HT-WLI with SIB can reliably deliver prescribed doses to heterogeneous and extensive thoracic lesions in EwS patients.

The key limitations of this study include the small sample size (n=5) and retrospective single-center design, which prevent any statistical inference, generalizability, and external validity. All procedures followed standard clinical protocols and validated assessment criteria (RECIST, QUANTEC), supporting the internal validity of observations; however, potential selection bias cannot be excluded. Additional limitations include respiratory motion management using abdominal compression and standardized 5-mm PTV margins rather than 4DCT/ITV-based individualized planning, short follow-up (median 5.5 months) that precludes assessment of long-term outcomes and late toxicities, and the inability to isolate the independent effect of HT-WLI due to concurrent systemic therapies. All findings are therefore considered hypothesis-generating.

### 5.2 Comparison with similar and contrasting cases

Our findings align with historical evidence supporting WLI in pulmonary metastatic EwS. Paulussen et al. (5) reported that WLI reduced lung recurrence (20% vs. 40%, p=0.046) and improved 5-year event-free survival (38% vs. 27%, p=0.0022) in 114 patients. Similarly, Scobioala et al. (12) found WLI combined with post-recurrence therapy improved 3-year PFS (36% vs. 14%, p=0.001) and OS (47% vs. 33%, p=0.007) in pulmonary relapsed EwS—consistent with our Case 4 achieving CR with WLI plus chemotherapy.

Our Case 2 (36 metastases achieving CR) contrasts with reports suggesting limited efficacy of WLI for extensive disease. Reiter et al. (11) found that only patients achieving rapid complete response of pulmonary metastases had improved outcomes, suggesting that chemosensitivity predicts WLI benefit. Our patient’s extensive disease (36 lesions) was chemorefractory (progressing on VDC/IE and VAIA) yet highly radiosensitive, highlighting that radiation response may diverge from chemotherapy response in EwS—an important clinical observation.

Our Case 5 (grade 3 RP with normal lung dosimetry) contrasts with typical radiation pneumonitis reports and aligns with emerging literature on checkpoint inhibitor pneumonitis (CIP) potentiation by radiotherapy. Azhar et al. (23) reported that thoracic radiotherapy increases CIP risk, and Bi et al. (24, 25) found that prior/concurrent immunotherapy increases RP risk, with stricter OAR constraints needed. Liu et al. (26) confirmed that thoracic radiotherapy combined with PD−1/PD−L1 inhibitors significantly increases pneumonitis risk, independent of conventional dosimetric parameters, supporting our observations in Case 5. The rapid response to steroids (3 days) is consistent with CIP management guidelines (27). This case adds to the limited literature on combined immunotherapy-radiotherapy in sarcoma, where Tawbi et al. (28) found no responses to pembrolizumab monotherapy in EwS, but radiotherapy may remodel the tumor microenvironment to improve immunotherapeutic sensitivity (29) and potentially enable abscopal effects (30). Zagardo et al. (31) demonstrated that radiotherapy can delay treatment changes in immunoresistant oligoprogressive disease, which helps explain the potential value of combining WLI with immunotherapy for progressive EwS.

### 5.3 Take-away lessons

#### 5.3.1 Lesson 1

HT-WLI-SIB is technically feasible across diverse metastatic presentations. The technique achieved excellent target coverage and OAR sparing in miliary disease (Case 2), pleural-only disease (Cases 1,3), thoracic radiotherapy delivered to spatially distinct, non-overlapping target volumes (Case 5), and multifocal metastatic disease with complex target geometry (Case 5). Dosimetric parameters (mean heart dose 7.39 Gy, lung V20 19.15%) compare favorably to historical AP/PA WLI (15, 16) and align with modern series using IMRT/VMAT (17–19).

#### 5.3.2 Lesson 2

Ewing’s sarcoma exhibits high radiosensitivity consistent with published evidence (20) that may be independent of chemosensitivity. Case 2 (36 lesions progressing on chemotherapy) achieved CR after WLI (12 Gy) plus sequential boost irradiation (15 Gy/5 fractions), supporting WLI even in chemorefractory disease. This has practical implications: patients with progressive pulmonary metastases on systemic therapy should not be denied potentially curative WLI.

#### 5.3.3 Lesson 3

Concurrent immunotherapy with WLI carries increased RP risk even with favorable lung dosimetric parameters. Case 5 developed grade 3 RP with lung Dmean 14.27 Gy and V20 3.9%—well below QUANTEC constraints (21). When combining WLI with immunotherapy, stricter lung dose constraints may be required, as emphasized in relevant clinical safety studies (24, 25, 27).

#### 5.3.4 Lesson 4

Comprehensive local irradiation of all identifiable metastatic lesions may improve long-term disease control and should be considered with rational treatment sequencing. Ahmed et al. (32) and Chang et al. (33) reported favorable survival outcomes in metastatic EwS patients receiving comprehensive radiotherapy for all detectable lesions. In our cohort, Case 2 obtained meaningful remission after whole-lung irradiation for extensive pulmonary metastases, while Case 5 achieved systemic lesion control via combining WLI with simultaneous integrated boost to thoracic metastases, followed by additional local irradiation to extra-thoracic metastatic lesions. This finding supports the individualized use of comprehensive local irradiation for eligible metastatic EwS patients.

#### 5.3.5 Lesson 5

Multidisciplinary management is essential. The varied concurrent therapies (anlotinib, sintilimab, VDC/IE) reflect real-world practice where WLI is integrated into ongoing systemic treatment. Close monitoring for interactions (e.g., RP with immunotherapy (24, 25, 27), myelosuppression with chemotherapy) is critical.

## 6 Patient perspective

We obtained written informed consent from all patients and interviewed them about their experience. Representative quotes:

### 6.1 Case 2 (15-year-old male, 36 metastases)

“When the doctor showed me the scan with so many spots in my lungs, I was terrified. But after treatment, they said all the spots were gone. I can breathe normally and play basketball again. The daily treatments were quick—just lying there for 15 minutes—and I didn’t feel anything. I’m grateful we did this.”

### 6.2 Case 5 (36-year-old female, grade 3 pneumonitis)

“The coughing started about a month after finishing radiation. It got scary—I couldn’t catch my breath walking to the bathroom. When they put me on steroids, I felt better within days. The doctors explained that combining the immunotherapy with radiation might cause this, but we all agreed it was worth it because the tumors in my chest disappeared. I’m back to normal now and have no regrets.”

### 6.3 Case 4 (25-year-old male, lung recurrence)

“Having cancer come back after two years of treatment was devastating. But the radiation to my whole lungs gave me hope again. The team explained how the machine would shape the radiation to avoid my heart and lumbar spine where I had radiation before. I finished treatment two months ago and my scans are clean. I’m planning to go back to work next month.”

## 7 Conclusions

Helical tomotherapy–based WLI with SIB appears feasible and adaptable across clinically challenging scenarios of pulmonary metastatic Ewing’s sarcoma, with promising short-term local control (86.7% ORR) and tolerable acute toxicity. The occurrence of grade 3 radiation pneumonitis in a patient with favorable lung dosimetry who received concurrent immunotherapy highlights the need for stricter OAR constraints when combining WLI with checkpoint inhibitors. As a small, preliminary case series, our findings are hypothesis-generating. Larger prospective studies with longer follow-up are needed to validate long-term safety and efficacy, and to optimize combination regimens with targeted and immunotherapy.

## Data Availability

The original contributions presented in the study are included in the article/supplementary material. Further inquiries can be directed to the corresponding authors.
